# Determination of Novel Anti-Cancer Agents by Targeting OGG1 Enzyme Using Integrated Bioinformatics Methods

**DOI:** 10.3390/ijerph182413290

**Published:** 2021-12-16

**Authors:** Ziyad Tariq Muhseen, Mustafa Hussein Ali, Nawar Rushdi Jaber, Dheyaa Shakir Mashrea, Ali Mamoon Alfalki, Guanglin Li

**Affiliations:** 1 School of Life Sciences, Shaanxi Normal University, Xi’an 710062, China; ziyad.tariq82@gmail.com; 2 Key Laboratory of Ministry of Education for Medicinal Plant Resource and Natural Pharmaceutical Chemistry, Shaanxi Normal University, Xi’an 710062, China; 3 School of Life Science and Technology, Huazhong University of Science and Technology, Wuhan 430074, China; m.bio24@yahoo.com; 4 Department of Medical Laboratory Techniques, School of Life Sciences, Dijlah University College, Baghdad 00964, Iraq; Biomoon.n@gmail.com; 5 Educational Directorate of Babylon Province, Ministry of Education, Babylon 51002, Iraq; dheyaashakir88@gmail.com; 6 Department of Health Professional Graduate, University of New England, Biddeford, Portland, ME 04005, USA; aalfalki@une.edu

**Keywords:** base excision repair, binding free energies, glycosylase inhibitor, MD simulations, molecular docking, TH5487

## Abstract

The **8**-oxoguanine DNA glycosylase (OGG1) enzyme is a key DNA glycosylase mediating the excision of 7,8-dihydro-8-oxoguanine (8-oxoG) from DNA molecule to the start base excision repair pathway. The OGG1 glycosylase function depletion has been seen to obstruct pathological conditions such as inflammation, A3 T-cell lymphoblastic acute leukemia growth, and neurodegenerative diseases, thus warranting OGG1 as an attractive anti-cancer enzyme. Herein, we employed several drug libraries intending to screen non-toxic inhibitory molecules against the active pocket of the enzyme that achieved stable binding mode in dynamics. Two anti-cancer compounds ([O-]C1=C(CC2=CC=CC=C2)SC(=[N+]1CC(=O)NC3=NC=C(CC4=CC=CC=C4)S3)S and CCCN(CCC)[S]-(=O)(=O)C1=CC=C(C=C1)C(=O)NNC2=NC3=CC=C(Br)C=C3C(=N2)C4=CC=CC=C4) from Selleckchem.com were identified to occupy the active pocket of OGG1 and bind with greater affinity than **Control** TH5487. The binding affinity of **Top-1** is −11.6 kcal/mol while that of **Top-2** is −10.7 kcal/mol in contrast to TH5487 **Control** (−9 kcal/mol). During molecular dynamic simulations versus time, the said compounds are tightly held by the enzyme with no minor structural deviations reported except flexible loops in particular those present at the N and C-terminal. Both the compounds produced extensive hydrophobic interactions with the enzyme along with stable hydrogen bonding. The docking and molecular dynamics simulations predictions were further validated by molecular mechanics with generalized Born and surface area solvation (MM/GBSA) and Poisson Boltzmann surface area (MM/PBSA), and WaterSwap binding energies that validated strong binding of the compounds to the enzyme. The MM/GBSA binding free energy for **Top-1** complex is −28.10 kcal/mol, **Top-2** complex is −50.14 kcal/mol) and **Control** is −46.91 kcal/mol while MM/PBSA value for **Top-1**, **Top-2** and **Control** is −23.38 kcal/mol, −35.29 kcal/mol and −38.20 kcal/mol, respectively. Computational pharmacokinetics support good druglike candidacy of the compounds with acceptable profile of pharmacokinetics and very little toxicity. All these findings support the notion that the compounds can be used in experiments to test their anti-cancer activities.

## 1. Introduction

Reactive oxygen species (ROS) are the product of exogenous and endogenous sources, the latter ROS are generated as a by-product of oxygen metabolism in the endoplasmic reticulum, mitochondria, and peroxisomes [1]. It has been well established that many pathological diseases such as aging, cardiovascular diseases, cancer, neurodegenerative diseases, inflammatory diseases, and ischemia-reperfusion injury result in loss of balanced redox homeostasis [2,3]. ROS in appropriate concentrations function in immune responses and signaling pathways; however, when ROS concentration reaches a higher level, they overwhelm the antioxidant potential of the cells, which in turn results in oxidative stress leading to oxidative damage to the DNA, protein, and lipids [3,4].

Under low redox conditions, guanine is sensitive to oxidation, transforming it to 7,8-dihydro-8-oxoguanine (8-oxoG) thus causing a genetic mutation [5]. In such a situation, 8-oxoguanine DNA glycosylase (OGG1) enzyme performed excision of 8-oxoG and along with PARP1 initiated base excision repair (BER) pathway, which has been involved in repairing the majority of DNA lesions [6]. Previous data revealed that availability of four approved PARP inhibitors to treat homologous recombination defective cancers [7]. These inhibitors interfere with alternative end-joining pathways [8] and replication forks [9]. The OGG1 enzyme of the BER pathway is less explored as an anti-cancer target. Recent studies indicated that inhibiting OGG1 by a potent small molecule, TH5487, SU0268 (https://www.medchemexpress.com/su0268.html (accessed on 8 December 2021)) and O8 (https://www.tocris.com/products/o8-ogg1-inhibitor_6236 (accessed on 8 December 2021)), effectively targets OGG1 enzyme and thus can serve beneficial approach for targeting oxidative DNA repair and alleviating cancer pathologies [10,11]. 

In the current study, considering the high potential of OGG1 as a druggable enzyme prompts us to devise a computational study to perform structure-based virtual screening of different natural and synthetic drug libraries with the ultimate aim to identify potent OGG1 inhibitors. OGG1 is a druggable enzyme as it can be targeted by drugs that can modulate its function. Computational tools in modern drug discovery play a major role in the development of therapeutically important drugs [12,13]. The in silico tools aid in the rational design of safe and new drugs and limit the use of animal models [14,15]. The study objectives include filtering several drug libraries including anti-cancer compounds (Selleckchem anti-cancer compound library I and II), plus libraries of natural compounds including medicinal plant database (MDP3) [16], Asinex targeted oncology database, and comprehensive marine natural products database (CMNPD), first on toxicity where toxic molecules were discarded. Toxic compounds offer failure at several stages of the drug discovery process and waste the invested costs, time, and human efforts [17,18,19]. Secondly, molecular docking was applied to virtual screen the mentioned non-toxic drug molecules against the OGG1 active pocket [20,21]. Further, the shortlisted best inhibitors were complexed with the enzyme and tested in dynamics conditions to evaluate binding conformation and binding interactions stability as a function of time. Extensive, all-atoms molecular dynamics simulation studies were performed for selected complexes [22,23] and intermolecular interactions energies were estimated by molecular mechanics with generalized Born and surface area solvation (MM/GBSA) and Poisson-Boltzmann surface area (MM/GBSA) methods [24,25], which were cross-validated by a further improved WaterSwap free energy method [26,27]. Overall, the study identified two novel drug molecules with good binding potential against OGG1 and therefore needs to be subjected to experimental studies.

## 2. Results

To evaluate the binding affinity of the filtered libraries’ compounds for the OGG1 enzyme, structure-based virtual screening was conducted. The active site information was retrieved from crystal structure of the enzyme reported with TH5487 inhibitor [10]. Performing virtual screening of the libraries containing approximately 46,283 molecules took about 1 month. The compound binding potential to the enzyme was measured first in terms of the GOLD fitness score that takes into account chemical forces build as a result docked conformation between the compound and the enzyme. A high GOLD fitness score determines better binding affinity of the compounds and vice versa. Top-ranked 10 molecules were redocked to the enzyme three times and each time the scoring function was changed to ASP (the Astex Statistical Potential), CHEMPLP, and ChemScore. Details about these scoring functions can be found on the GOLD docking software official page (https://www.ch.cam.ac.uk/computing/software/gold-suite(assessed on 8 December 2021). **Top-1**0 docked inhibitors at the active pocket of the receptor enzyme predicted by GOLD docking software are shown in Figure S1. It can be easily observed that these virtually screened compounds are docked well inside at the active pocket of the enzyme. In parallel, **Top-1**0 compounds binding energy was determined using AutoDock Vina to strengthen our docking predictions. Two compounds ([O-]C1=C(CC2=CC=CC=C2)SC(=[N+]1CC(=O)NC3=NC=C-(CC4=CC=CC=C4)S3)S and CCCN(CCC)[S](=O)(=O)C1=CC=C(C=C1)C(=O)NNC2=NC3-=CC=C(Br)C=C3C(=N2)C4=CC=CC=C4) were selected as the best inhibitors of the enzyme compared to the **Control** TH5487, SU0268, and O8. The GOLD fitness scores of **Top-1** and **Top-2** are 79 and 76, respectively, while their AutoDock Vina binding energy is −11.6 kcal/mol and −10.7 kcal/mol, respectively. In case of negative **Control**s, the binding affinity of TH2840 or TH5411 was −8.61 kcal/mol and −8.01 kcal/mol, respectively. The different docking scores of the **Control** and compounds are tabulated in Table 1. 

**Figure 1:**
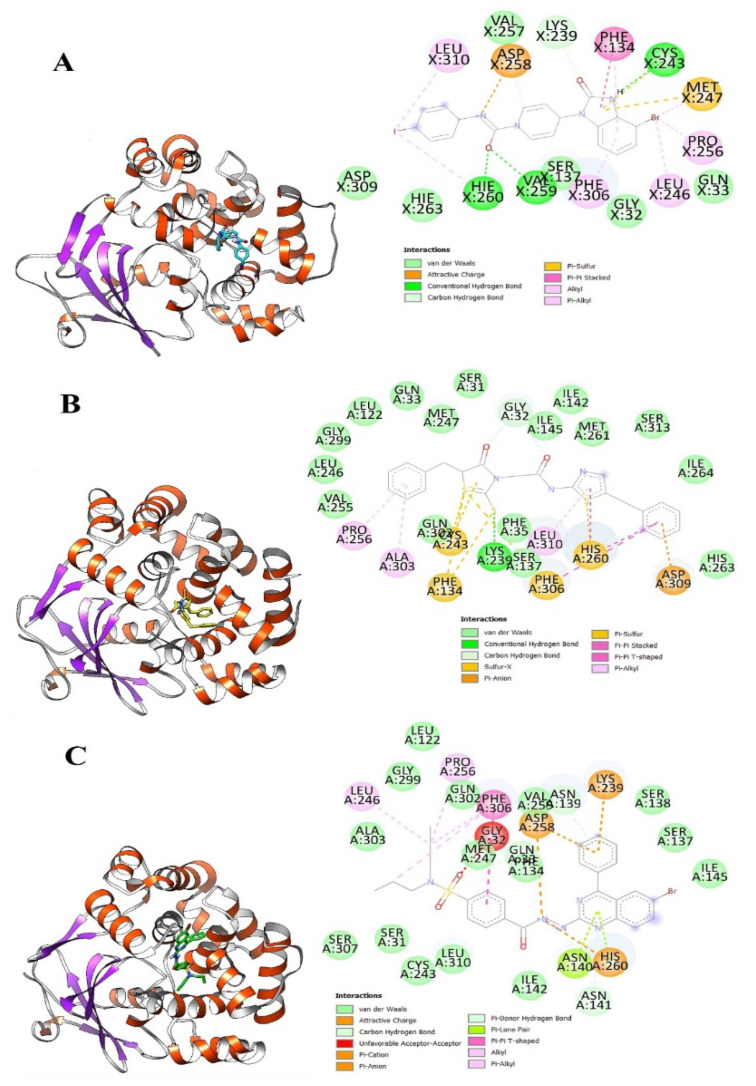
Molecular docking analysis of **Control** and shortlisted best binders. Both intermolecular docking conformation and interactions are provided. (**A**) 3D conformation of **Control** (shown in cyan stick), (**B**) **Top-1** (shown in yellow stick), and (**C**) **Top-2** (shown in green stick) at the active pocket of OGG1 enzyme (shown in secondary structure ribbon), and 2D presentation of enzyme residues interaction with the compounds.

The **Control** TH5487 molecule’s GOLD fitness score is 72 and its binding energy value is −9.0 kcal/mol. Both hydrogen bonding and van der Waals interactions are reported at the active pocket by the **Control**. Cys243, Val259, and Hie260 are hydrogen bonding residues with distance lengths of 4.02 Å, 3.8 Å, and 4.54 Å, respectively. While Gly32, Gln33, Lys239, Val257, Asp309, Hie263, and Gly32 formed van der Waals interactions with the compound. Besides these, Phe134, Leu246, Pro256, Phe306, and Leu310 were noticed in different alkyl interactions. Interestingly, the docked site of the compounds and the **Control** remain the same and interact with an almost similar set of active pocket residues. The binding mode of **Control** and its interactions with the active site residues are shown in Figure 1A. 

**Top-1** interactions at the docked active cavity are dominated by van der Waals. These interactions are formed along the length of the compound where the central oxygen and nitrogen atoms are engaged by hydrogen bonding in particular with Lys239 (distance length is 2.3 Å). The end benzene on one side produced interactions of van der Waals and pi-anion while the on the other side make pi-alkyl and van der Waals. The central sulfur atoms of the compounds interact with Cys243, and Phe134 through sulfur bonding. From all around, the compound is strongly held at the active pocket and favors interactions with majority of the active pocket residues (Figure 1B). A rich cluster of chemical interactions is also noticed between **Top-2** compound and the receptor enzyme (Figure 1C).

### 2.1. Molecular Dynamics Simulations

**Figure 2:**
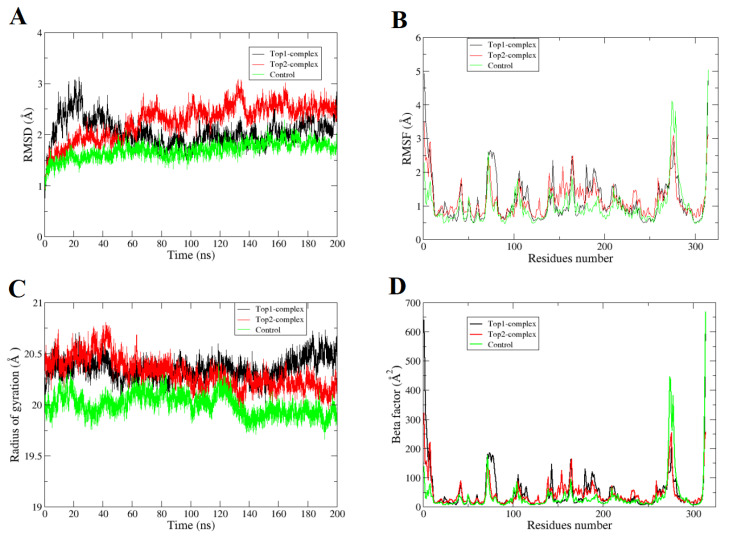
Molecular dynamic simulation analysis. All provided analysis are done based on carbon alpha atoms. The structural dynamics of complexes are given as a function of time. (**A**) RMSD, (**B**) RMSF, (**C**) Rg, and (**D**) B-factor.

Molecular dynamic simulation studies determine the dynamic behavior of atoms or macromolecules in a specific time and special environment. Both selected complexes (GOLD docked pose) and **Control** were performed to validate structural stability of the enzyme in the presence of compounds. This was also essential to look for stable binding of the compounds at the docked site throughout the length of simulation time, which is key for altering the biological function of the enzyme and to get desired results [22]. The simulation trajectories were evaluated through several statistical parameters (Figure 2). First, root mean square deviation (RMSD) [28,29] was performed that examined all carbon alpha deviation versus time scale considering the initial input intermolecular conformation as reference (Figure 2A). Lower RMSD implies fewer deviations in the system, whereas higher RMSD corresponds to more structural fluctuations. Larger biological systems usually follow higher RMSD, but a constant trend indicates their stable nature. 

The Figure 2A shows that compounds are in good equilibration and the stability is more strongly towards the end of simulation time. The **Control** was reported with an excellent RMSD plot and is a demonstration of greater intermolecular strength. The mean RMSD of **Top-1**, **Top-2**, and **Control** complexes is 1.68 Å, 2.04 Å, and 2.27 Å, respectively. The **Top-1** compound enzyme is seen as not stable until 52 ns, as it experienced deviations touch 3.1 Å. Following this, the system remained in stable RMSD until the end of simulation time. **Top-2** compound had a lower RMSD until 60 ns and then behaved inconsistently with maximum RMSD of 3 Å, and after 140 ns the system became stable. These acceptable RMSD variations upon snapshots visualization identified that compounds are trying to stabilize their binding conformation at the active pocket by giving flexibility to some of its chemical moieties, which forces the enzyme flexible loops to lose their original XYZ coordinates [30,31]. To validate the stability of the systems, the simulation was run in duplicate with a different initial velocity. A very similar trend was noticed except for few up and down trends (Figure S2). To infer time dependent enzyme residue fluctuations in the presence of inhibitors, root mean square fluctuation (RMSF) was calculated (Figure 2B). The mean RMSF of the systems is as **Top-1** complex (1.16 Å), **Top-2** complex (1.15 Å), and **Control** (0.99 Å). These RMSF values agree on high stable nature of the complexes, in particular the **Control**. Higher RMSF values were recorded for the N-terminal and C-terminal residues covering large loops; they are more flexible than the rest of the enzyme regions. The active pocket residue remained highly stable in the presence of the compounds. The radius of gyration (Rg) [32] was also taken into consideration as given in Figure 2C. Rg is a statistical value describing the strength of atoms packing in a protein. The lower value and the decline in the Rg value for all complexes is suggestive of stabilization and compactness aided by the binding of ligands to the target protein. The mean Rg of systems is ~20 Å and follows the same trend as noticed in RMSD. Beta factor (B-factor) analysis [33] was done to investigate thermal stability of the enzyme residues (Figure 2D). This analysis replicates the RMSF findings and confirmed the C and N-terminal have more flexibility than the rest of the enzyme structure. To validate the binding site stability of the compounds, a superimposition of last frame of molecular dynamics simulation over docked enzyme-compound structure was performed as can be seen in Figures S3 and S4. In both cases, it was noticed that the compounds occupied the binding pocket until the end of simulation time and remained in contact with the enzyme active site residues. 

### 2.2. Hydrogen Bonds Analysis

**Figure 3:**
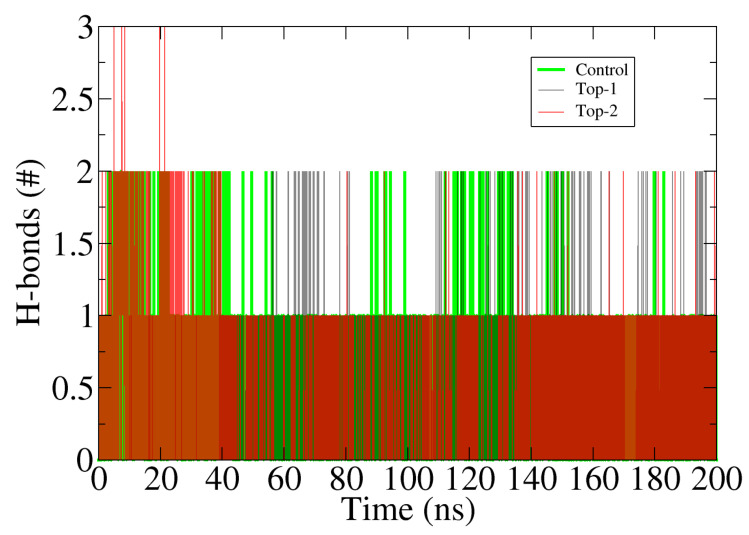
Hydrogen bonds analysis in each frame of simulation time. The bar figure provides number of hydrogen bonds established between the compounds and enzyme during simulation time.

In drug designing against any particular biological macromolecule, hydrogen bonding plays a significant role in three ways [34]. First, it stabilizes the binding of a compound to the binding receptor partner. Second, it aids in chemical recognition of the compounds, and third, it determines compound binding affinity, which is key to success in drug development. Throughout the the simulation, the number of hydrogen bonds formed by both compounds as well **Control** is presented in Figure 3. As can be seen, the compounds and **Control** are strongly engaged by at least one hydrogen bond in each frame of simulation trajectories. This signifies the high intermolecular affinity of the docked compounds and OGG1 enzyme. These hydrogen bondings were seen between highly electronegative atoms of the compounds/**Control** and close distance active site residues presented in RDF analysis. 

### 2.3. Radial Distribution Function-g(r)

**Figure 4:**
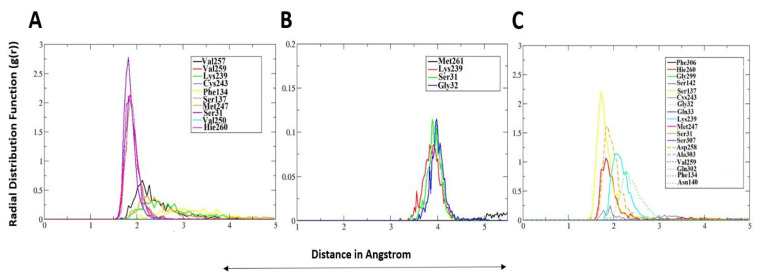
Radial distribution analysis of hydrogen bonds/van der Waals formed between the compounds and enzyme active site residues. (**A**) **Control**, (**B**) **Top-1**, and (**C**) **Top-2**. Only key interactions are plotted in radial distribution analysis.

Determining interatomic interaction density during simulation time is important in several ways for stable interactions of the compounds with the enzyme [35]. It helps in highlighting constant interactions vital for holding compounds at the enzyme docked site and shedding light on the distance at which maximum interactions density is produced [36]. Before running g(r) analysis, an in-house Perl written script was used in visual molecular dynamics (VMD) [37], which was run on all simulation trajectories loaded on systems prmtop. The close distance hydrogen bonds and van der Waals bonds between the compounds and the OGG1 enzyme were filtered and subjected Assisted Model Building with Energy Refinement (AMBER) CPPTRAJ to generate g(r) plots. Only interactions that are important in ligand binding to the enzyme active pocket and regularly seen during simulation frames were selected for RDF analysis. For **Control**, residues like Ser31, Phe134, Ser137, Lys239, Cys243, Met247, Val250, Asp258, Val257, and Hie260 were seen in regular contacts with the **Control** O2, and H7 atoms throughout simulation time. At different time points, the g(r) plots describing the mentioned residues interactions with the **Control** atoms are presented in Figure 4A. The maximum g(r) reported for Ser137 oxygen atom to **Control** H7 and Hie260 and **Control** O2 atom towards the end of simulation. This means that at the end of simulation time, these two interactions are key stable binding of the **Control** and play a critical contribution in the initial times. For **Top-1**, Ser31, Gly32, Lys239, and Met261 are vital in interactions with the compound’s highly electronegative atoms in particular oxygen and nitrogen (Figure 4B). The Ser31, Gly32, and Lys239 interaction density with **Top-1** atoms were reported maximum at distance ~4 Å. In case of **Top-2**, enzyme active pocket residues like Ser137, Asp258, Lys239, His260, Asn140, and Ile142 are among the notable residues engaging the compound (Figure 4C). Ser137, Asn140, Asp258, and Hie260 were found to have highest interatomic interaction density distribution at ~1.8–1.9 Å.

### 2.4. MM/GBSA and MM/PBSA Binding Free Energies

Next, binding free energies of the complexes were estimated using two popular approaches: MM/GBSA and MM/PBSA. Both approaches are now routinely applied in many drug designing works to decipher compounds real binding potential. The advantage of these end point techniques is their low computational power need and their potential to generate reproducible results comparable to experimental findings [38,39]. In the MM/GBSA method, the total binding energy of the complexes is the same as **Top-1** complex (−28.10 kcal/mol), **Top-2** complex (−50.14 kcal/mol) and **Control** (−46.91 kcal/mol). The MM/GBSA has ranked compound 2 as the most effective binder of OGG1 enzyme, followed by **Control** and **Top-1**. For all three complexes, van der Waals energy was seen dominant while electrostatic energy favors the binding. As a result of significant contribution from both van der Waals and electrostatic energy, the net gas phase of the systems is quite promising and denotes the systems overall equilibrium nature. On the other hand, net solvation energy is non-favorable mainly due to polar solvation energy. The non-polar energy seems to contribute to systems stability. In MM/PBSA, the **Control** complex is a bit more stable than compound 2 and compound 1. Similarly, MM/GBSA, MM/PBSA also interpreted the domination of van der Waals and electrostatic energies while highly non-favorable contributions were derived from polar solvation energy. The total MM/PBSA energy of **Top-1**, **Top-2** and **Control** is −23.38 kcal/mol, −35.29 kcal/mol and −38.20 kcal/mol, respectively. The different binding free energies of the complexes are tabulated in Table 2. The entropy energy for systems is **Control** (10 kcal/mol), **Top-1** (11 kcal/mol) and **Top-2** (8 kcal/mol). These findings complement the aforementioned analysis well in demonstrating the good binding capacity of the compounds for the OGG1 enzyme, and are likely to compete with natural ligands to block the enzyme activity and prevent cancers. 

### 2.5. Decomposition of MM/GBSA Binding Energy 

The net MM/GBSA binding free energy was decomposed into the enzyme residues to shed light on the hotspot residue contributing majorly to binding with the compounds during simulation time [26,40]. The different residues that are involved in regular binding with the compounds and highly stable in complex formation are tabulated in Table 3. In both complex, the binding energy of ligand molecules is much lower than predicted by the AutoDock Vina and are therefore achieving greater stability when binding to the receptor enzyme. Majority of the hotspot residues in both complexes are part of the OGG1 enzyme active pocket. The residues interact hydrophobically and hydrophilically with the compounds. 

### 2.6. WaterSwap Binding Energies 

**Figure 5:**
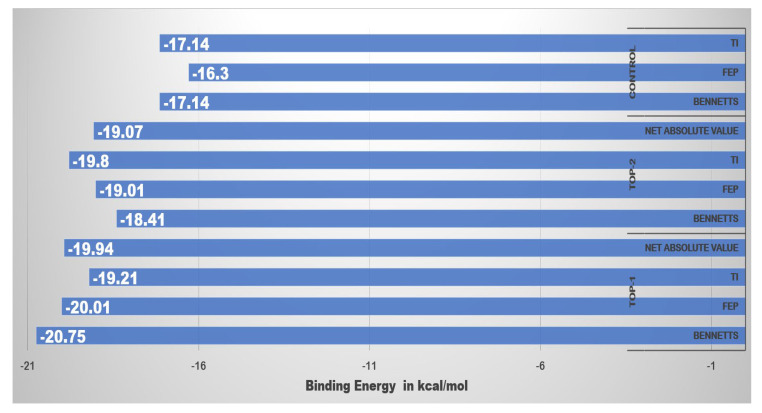
WaterSwap binding free energies are estimated by different methods such TI, FEP, and BENNETTS. The energy values are given in kcal/mol.

Though there are several advantages of using MM-GBSA and MM-PBSA binding energy methods in drug designing process, yet they suffer from a few limitations that are critical to be considered. Among the most important is skipping the role of water molecules present in the enzyme active pocket especially in cases when the water molecules bridge the interactions between receptor and ligand [26]. To overcome this, we employed another popular binding free energy method in the work to get more confidence in our results. WaterSwap allows swapping of a ligand with an equal volume of water and thus is more sophisticated in terms of considering water molecules contribution in ligand binding. WaterSwap uses three algorithms namely, thermodynamics integration (TI), free energy perturbation (FEP), and BENNETTS to calculate binding free energy of a given complex. As can be noticed in Figure 5 the total binding energy value by each WaterSwap method is very converged with respect to each other and the values are quite low demonstrating highly stable binding of the compounds to the enzyme. 

### 2.7. In Silico Site Directed Mutagenesis

In order to access the impact of mutations on the active pocket to bind compounds, site directed mutagenesis analysis was performed. It has been revealed that several residues are affecting stable binding conformation of the compounds upon mutation. The binding free energy of residues which is decreased after mutation is presented in Table 3. It is also noted that the binding proximity and conformation of the compounds remain the same; however, the binding strength is reduced due to absent of active residues chemical moieties which were present in the unmutated enzyme. These results supported the fact that enlisted residues are vital in compound binding, interactions, and overall conformation stability at the enzyme active pocket.

### 2.8. Computational Pharmacokinetics Studies

Computational predictions of the inhibitors’ pharmacokinetics were carried out to guide structural optimization of the compounds to avoid their failure in clinical studies [41,42,43]. The results of this analysis are given in Table 4. Both compounds are moderately soluble thus have good chances to be easily administered orally. The physicochemical properties of the compounds are favorable and thus can be good drug candidates. The topological polar surface area (TPSA) [44] of the compounds is within acceptable range, which can be improved further, in particular **Top-1**, to enhance their cell permeability potential and reach the target site for performing desired biological mechanisms. Fortunately, both compounds are much less toxic as per predictions, which makes them suitable against the targeted OGG1 enzyme. The compounds are predicted to show no hepatotoxicity, no Ames toxicity, and no skin sensitization, all in favor of the compounds to be utilized in further experimental testing against OGG1. Additionally, due to the simple structure of the compounds, they can be easily synthesized in bulk quantities and have no Pan-assay interference compounds (PAINS) alerts [45]. Because of good pharmacokinetic profiles of the compounds, they might serve as attractive drug molecules for blocking cancer cell proliferation. The compounds are also predicted to be non-toxic. 

## 3. Materials and Methods

### 3.1. OGG1 Structure Retrieval and Preparation for Virtual Screening

For the virtual screening, the complex OGG1-TH5487 was fetched from a protein data bank [46] (PDB-ID: 6RLW) [10] in UCSF Chimera 1.15 [47] and processed to prepare the OGG1 enzyme for virtual screening. The enzyme structure is determined by X-RAY diffraction method with a resolution of 2 Å, R-value free of 0.274, and R-value work of 0.223. All water molecules and co-crystallized TH5487 inhibitor were deleted from the structure except those present at the active pocket. The co-crystalized TH5487 inhibitor interactions with OGG1 enzyme are presented in Figure S5A. Subsequently, the structure was subjected to Dock Prep module of UCSF Chimera to add missing hydrogen atoms, charges, and complete missing side chains using Dunbrack 2010 rotamer library. The enzyme structure was then energy minimized through the Minimize structure module of UCSF Chimera where the steepest descent and conjugate gradient algorithms were run for 1000 cycles each. During this minimization process, the step size was kept at default of 0.02 Å. All standard residues of the enzyme were treated using an AMBER ff14SB force field [48] while charge addition used the Gasteiger method. The post-OGG1 enzyme structure was then loaded into PyRx 0.8 software [49] and converted into PDBQT (Protein Data Bank, Partial Charge (Q), & Atom Type (T)).

### 3.2. Inhibitors Library Preparation

Different libraries of anti-cancer compounds (Selleckchem anti-cancer compound library I (3547 compounds) and II (921 compounds)) plus libraries of natural compounds including medicinal plant database (MDP3) (~5000 compounds) [50], Asinex targeted oncology database (4815 compounds), and comprehensive marine natural products database (CMNPD) (~32,000 compounds) [51] were retrieved from their respective websites and filtered for non-toxic compounds in Discovery Studio 3.5 toxicity prediction module [52]. This filtration was important as toxicity is one of the leading causes among others resulting in failure of drugs during the development process [53]. The filtered molecules of each library were then imported to PyRx 0.8 [49] one by one where they were energy-minimized and converted to PDBQT.

### 3.3. Molecular Docking for Inhibition Studies

The docking process was done in Genetic Optimization for Ligand Docking (*GOLD) 5.2* [54] and the same set of parameters were used in AutoDock Vina [55]. In GOLD, different scoring functions like ASP, CHEMPLP, and CHEM SCORE were considered. For comparative molecular docking, we utilized AutoDock Vina of PyRx 0.8 to carry out binding studies of all shortlisted drugs against OGG1 enzyme. The grid was box of size (15 × 15 × 15 along with the XYZ) was set around Gly32 oxygen atom to guide binding of the libraries’ molecules. Each molecule was allowed to generate 20 conformations at the defined OGG1 active pocket. The GOLD results were then sorted in ascending order and the highest GOLD score conformation of the molecules was selected and complexed with the enzyme. For validation, the co-crystalized TH5487 molecule, and two other **Control**s like SU0268 and O8 were docked to the receptor enzyme using the same procedure and the output conformation as compared to the original to get affirmation on the docking procedure. The docked TH5487 chemical interactions with pre-energy minimized OGG1 enzyme are given in Figure S5B. For negative **Control**, TH2840 or TH5411 were used [https://www.ncbi.nlm.nih.gov/pmc/articles/PMC6645780/] (8 December 2021). The top 10 solutions of GOLD were compared, and the best common binders were selected for downward computational studies. 

### 3.4. Molecular Dynamics Simulation Study 

For the molecular dynamic simulations, we employed AMBER20 software package [56]. Force fields like ff14SB force field were applied to generate parameters of the OGG1 whereas GAFF force field [57] and AM1-BCC atomic charges were run for ligands parameterization. The systems solvation was conducted in TIP3P water model of size 12.0 Å and then NaCl mediated neutralization at the concentration of 0.10 M was performed. As an example, the water box solvating **Control** complex is presented in Figure S6. Energy of the systems was minimized through 1500 steps of steepest gradient and 1500 steps of conjugate gradient methods. Each system was heated for a time scale of 50 ps keeping the temperature constant (300 K). Equilibration of the systems was then achieved for 50 ps using periodic boundary conditions considering constant pressure and Langevin thermostat [58]. Following systems equilibrium, NPT ensemble was used to conduct molecular dynamic simulation for a time scale of 100 ns with a temperature of 310 K. To constrain the bonds at their equilibrium lengths, we used the SHAKE algorithm [59]. To define van der Waals interactions, a cut-off value of 10-Å cut-off was considered, and a PME method [60] was employed for defining the electrostatic forces. Subsequently, for understanding systems dynamics different AMBER modules were used including CPPTRAJ [61] and MMPBSA.py [62]. For frame visualization at different nanoseconds, UCSF Chimera [47] and Discovery Studio v21.1.0.20298 [52] were utilized. Radial distribution function (RDF) plots were generated for chemical interactions that play a significant role in anchoring the compound at the docked site and increase intermolecular stability [35,63]. 

### 3.5. Estimating MM/GBSA and MM/PBSA Binding Free Energies 

To determine both total binding free energy and per-residue decomposition of selected complexes MM/GBSA and MM/PBSA approaches were used [24]. This was accomplished by running MMPBSA.py module of AMBER20 on all simulated trajectories of the systems. For the protocol, 500 frames from each system trajectories were picked and processed through the following equation,

∆G binding = ∆G enzyme-compound complex − [∆G enzyme + ∆G compound]

The entropy energy of each complex was determined using a bash script applied in a previous study [64]. 

### 3.6. WaterSwap Binding Energy Predictions

The docked complexes were then subjected to WaterSwap based binding energy predictions [26,27]. WaterSwap from the Sire package was used to calculate binding free energy for default 1000 iterations considering 25 million moves of Monte Carlo sampling across different 16 λs at pressure 1 atm and temperature 198.15 K for each replica. The binding free energy was investigated using three different algorithms: thermodynamic integration (TI), free energy perturbation (FEP), and Bennett’s acceptance ratio (BAR). Deviation in the net energy value of 1 kcal/mol indicates a good degree of systems convergence [65].

### 3.7. In Silico Site-directed Mutagenesis 

Alanine scanning analysis was further performed for OGG1 enzyme residues that are seen consistently important for interactions with the compounds [40]. Key residues of OGG1 enzyme were manually mutated to Alanine and the binding energy of the enzyme-compounds complexes was re-estimated using AMBER MM/GBSA method. The binding energy difference between native and mutants are termed as ΔΔ*G_bind_* and calculated as:

ΔΔGbind = ΔG binding energy of native Type − ΔG binding energy of mutants

More stability of the mutants can be inferred by a lower Δ*G_bind_* value and vice versa. 

### 3.8. Predictions about Compounds Pharmacokinetics 

In silico predictions about compounds, pharmacokinetics were done using online SWISSADME [42] and pkCSM servers [41].

## 4. Conclusions

Computer aided drug designing (CADD) techniques have been useful in discovery, developing and analyzing biological active drugs [12]. Compared to experimental drug designing, CADD significantly shortens the drug development time, reduces extra cost and increases the chances of successful design of new drugs. In this study, different approaches of CAAD are utilized to identify inhibitory molecules against the OGG1 enzyme, which is a potential target for treating cancer. As a result of structure-based virtual screening of both natural and synthetic compounds libraries against the enzyme, two drug molecules; ([O-]C1=C(CC2=CC=CC=C2)SC(=[N+]1CC(=O)NC3=NC=C(CC4=CC=CC=C4)S3)S and CCCN(CCC)[S](=O)(=O)C1=CC=C(C=C1)C(=O)NNC2=NC3=CC=C(Br)C=C3C(=N2)C4=CC=CC=C4) from Selleckchem library were identified as high affinity inhibitory molecules. Both the molecules are showing stable interactions with the enzyme active pocket residues and accomplished stable binding pose as the simulation time proceeds. The compounds strong binding to the enzyme is supported by multiple hydrogen bonds that anchor the compounds in the docked pocket. The enzyme-compounds complexes atomic level interactions are dominated by both electrostatic and van der Waals energies and revealed good systems equilibrium. Moreover, the compounds have an acceptable pharmacokinetics profile thus making them good candidates to be considered in additional structural optimization to get desired biological activity. The compounds are of different chemical nature than those reported previously and have shown stronger binding and well fitting inside the OGG1 pocket. Though these computational findings are promising, yet experimental validation to uncover true biological potency of the compounds to block the function of OGG1 enzyme still needed to be performed.

## Figures and Tables

**Table 1:** Virtually screened compounds and their docking scores.

Compound	GOLD Score	Autodock Vina Binding Free Energy (kcal/mol)	ASP	CHEMPLP	Chem Score
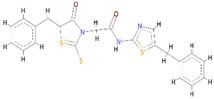 **Top-1**	79	−11.6	18.71	56.88	17.84
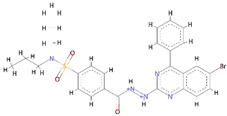 **Top-2**	76	−10.7	16.10	54.89	16.20
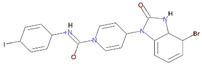 **Control**	72	−9.0	16.30	51.9	14.84
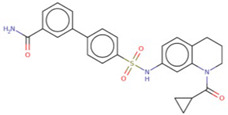	74 SU0268	−9.8	17.37	50.74	15.87
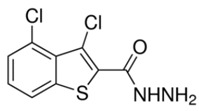	O8	−11.54	19.57	53.97	17.52

**Table 2:** MM/GBSA and MM/PBSA binding free energy estimated from 500 frames of molecular dynamics simulation. The net binding free energy value is decomposed further into respective electrostatic, van der Waals, polar, and non-polar solvation energies.

MM/GBSA							
Compound	ΔG Binding (kcal/mol)	ΔG Electrostatic (kcal/mol)	ΔG Bind van der Waals (kcal/mol)	ΔG Gas Phase (kcal/mol)	ΔG Polar Solvation (kcal/mol)	ΔG Non-Polar Solvation (kcal/mol)	ΔG Solvation (kcal/mol)
**Top-1**	−28.10	−25.26	−41.42	−66.69	44.40	−5.80	38.59
**Top-2**	−50.14	−17.00	−60.47	−77.47	34.54	−7.21	27.32
**Control**	−46.91	−23.10	−56.70	−79.81	38.54	−5.64	32.90
**MM/PBSA**							
**Top-1**	−23.38	−25.26	−41.42	−66.69	47.85	−4.54	43.30
**Top-2**	−35.29	−17.00	−60.47	−77.47	47.50	−5.32	42.17
**Control**	−38.20	−23.10	−56.70	−79.81	45.55	−3.94	41.60

**Table 3:** Net binding energy value of compounds and their interacting residues that are highly stable in complex formation in simulation time. The energy values are given in kcal/mol. Moreover, alanine scanning results when key compounds interacting residues are mutated to alanine. NA (not applicable).

Ligand/Residue	Top-1	Alanine Scanning Results	Top-2	Alanine Scanning Results
Ligand	−15.06	NA	−24.28	NA
Ile142	−3.209	−2.14	−4.38	−2.80
Phe134	−1.90	−1.20	−2.74	−1.11
Phe306	−1.77	−0.98	−2.84	−1.44
Ala143	−1.19	−1.0	−1.75	−1.12
Cys243	−0.97	NA	−1.55	NA
Gln33	−0.82	NA	−1.50	NA
Ile145	−0.80	NA	−1.35	NA
Met247	−0.71	NA	−1.28	NA
His260	−0.47	1.0	−1.10	−1.00
Leu122	−0.45	NA	−0.87	NA
Ala303	−0.41	NA	−0.80	NA
Leu246	−0.39	NA	−0.73	NA
Gly32	−0.35	−1.04	−0.70	−1.10
Pro256	−0.33	−1.0	−0.61	−1.01
Leu310	−0.25	NA	−0.55	NA
Phe35	−0.22	0.12	−0.54	−0.41
Val240	−0.21	NA	−0.50	NA
Ser31	−0.21	0.11	−0.48	1.18

**Table 4:** Computational pharmacokinetics along with several pharmaceutically important parameters of the compounds.

Property	Compounds		
Physicochemical Properties	Top-1	Top-2	Control
Formula	C22H19N3O2S3	C27H28BrN5O3S	C19H19BrIN4O2
Molecular weight	453.60 g/mol	582.51 g/mol	542.19 g/mol
Num. heavy atoms	30	37	27
Num. arom. heavy atoms	22	22	11
Fraction Csp3	0.14	0.22	0.32
Num. rotatable bonds	8	11	4
Num. H-bond acceptors	3	6	2
Num. H-bond donors	1	2	2
Molar Refractivity	123.71	149.71	118.21
TPSA	164.21 Å^2^	112.67 Å^2^	70.77 Å^2^
Lipophilicity			
Consensus Log Po/w	4.22	5.00	2.38
Water Solubility	Moderately soluble	Moderately soluble	Moderately soluble
Pharmacokinetics			
GI absorption	High	High	High
BBB permeant	No	No	Yes
P-gp substrate	No	No	Yes
CYP1A2 inhibitor	No	No	No
CYP2C19 inhibitor	Yes	Yes	No
CYP2C9 inhibitor	Yes	Yes	No
CYP2D6 inhibitor	No	No	No
CYP3A4 inhibitor	No	No	No
Log Kp (skin permeation)	−4.93 cm/s	−4.80 cm/s	−7.34 cm/s
Druglikensess			
Lipinski	Yes; 0 violation	Yes; 1 violation: MW > 500	Yes; 1 violation: MW > 500
Medicinal Chemistry			
PAINS	0 alert	0 alert	1 alert acyl_het_A
Synthetic accessibility	3.86	3.72	4.76
Toxicity			
Hepatotoxicity	No	Yes	Yes
Skin sensitisation	No	No	No
T. pyriformis toxicity	0.292 log ug/L	0.285 log ug/L	0.315 log ug/L
Ames toxicity	No	No	No
Minnow toxicity	0.69 log mM	−1.164 log mM	−1.14 log mM
Carcino mouse	No	No	No
Excretion			
Total clearance	0.763 log mL/min/kg	0.026 log mL/min/kg	0.031 log mL/min/kg
Renal OCT2 substrate	No	No	No

## Data Availability

The data presented in this study are available within the article.

## Supplementary Materials

**Figure S1**. Binding mode of top 10 hits at the active pocket of OGG1 enzyme predicted using GOLD. **Figure S2**. Duplicate RMSD of the systems with a different initial velocity. **Figure S3**. Superimposition of last frame of molecular dynamics simulation over docked enzyme-compound structure depicting stability of **Top-1** binding. **Figure S4.** Superimposition of last frame of molecular dynamics simulation over docked enzyme-compound structure depicting stability of **Top-2** binding. **Figure S5.** Co-crystalized TH5487 chemical interactions (A) and docked TH5487 chemical interactions. **Figure S6.** Submerged **Control**—OGG1 complex (surface blue magenta) in TIP3P water box.
