# Supplementary material for: Determination of Novel Anti-Cancer Agents by Targeting OGG1 Enzyme Using Integrated Bioinformatics Methods
Source: Int J Environ Res Public Health. 2021 Dec 16;18(24):13290. doi: 10.3390/ijerph182413290 (PMC8706639; doi:10.3390/ijerph182413290)
Supplement: Supplementary file 1 [file ijerph-18-13290-s001.zip › ijerph-1464150-supplementary.pdf]

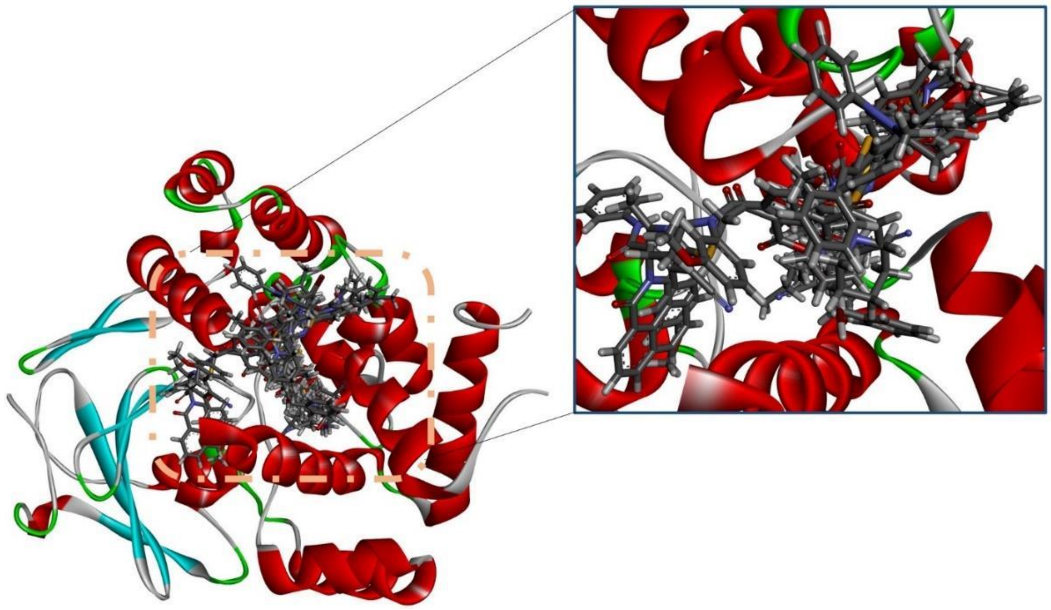

Figure S1. Binding mode of top 10 hits at the active pocket of OGG1 enzyme predicted using GOLD.

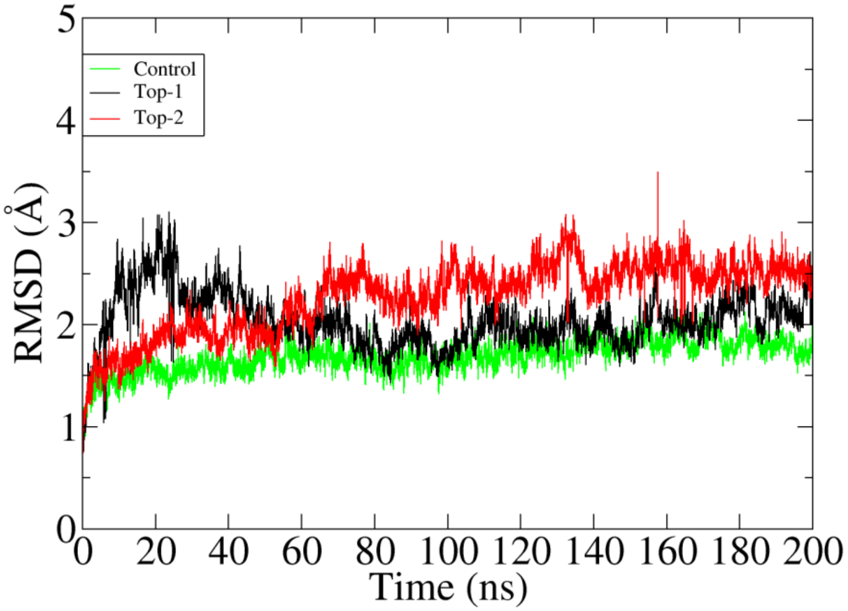

Figure S2. Duplicate RMSD of the systems with a different initial velocity.

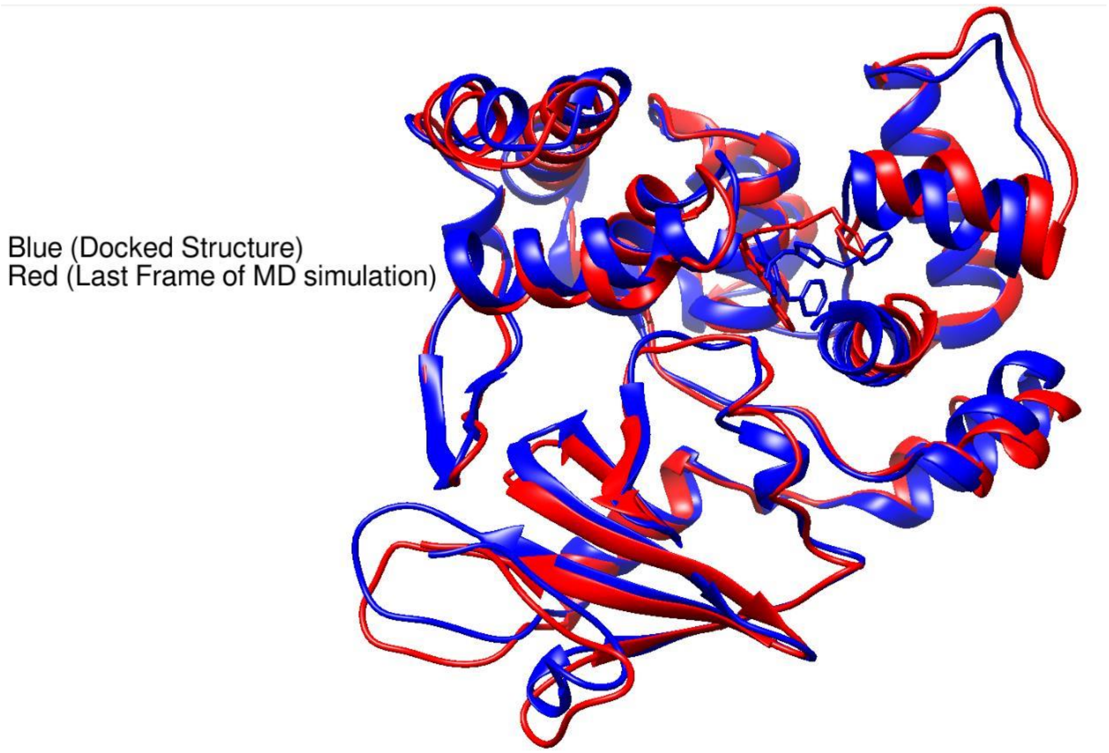

Figure S3. Superimposition of last frame of molecular dynamics simulation over docked enzyme-compound structure depicting stability of compound 1 binding.

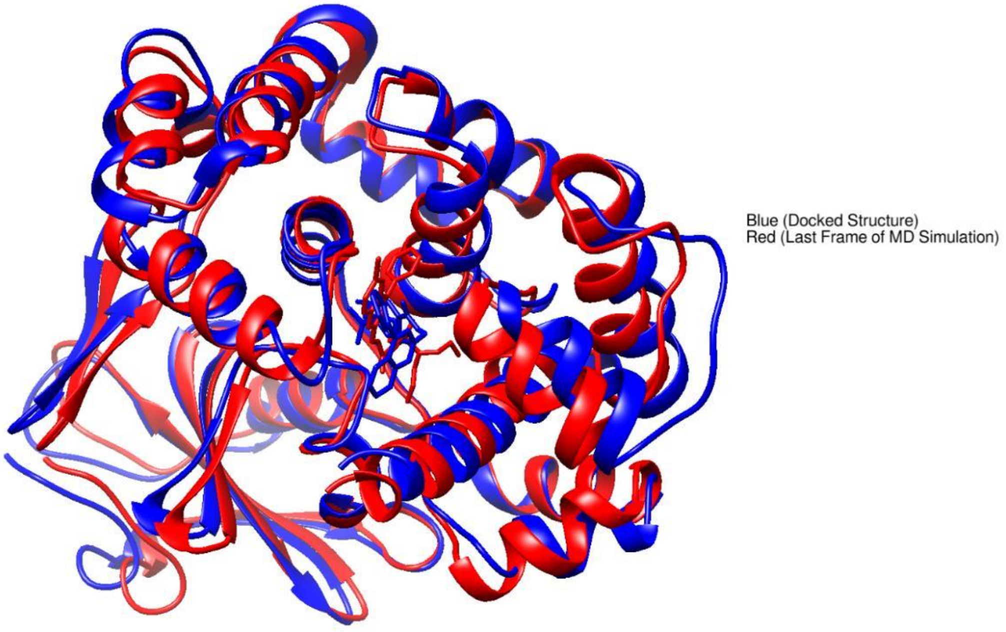

Figure S4. Superimposition of last frame of molecular dynamics simulation over docked enzyme-compound structure depicting stability of compound 2 binding.

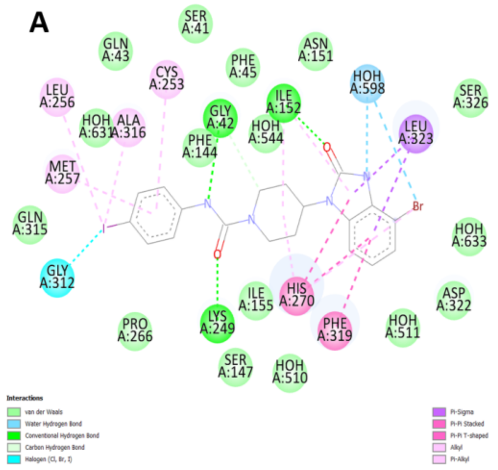

Co-crystalized TH5487 with OGG1 enzyme.

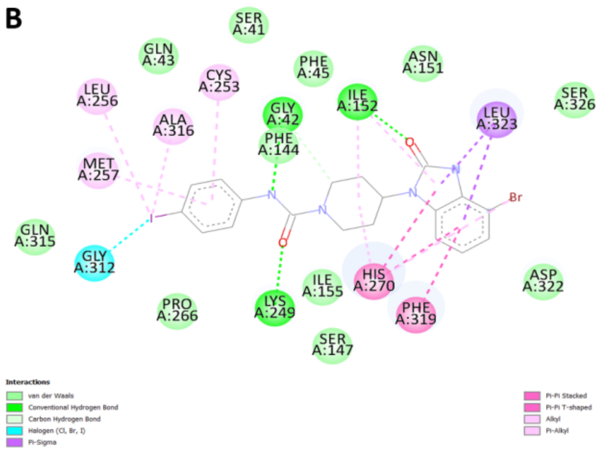

Docked TH5487 with pre-energy minimized OGG1 enzyme.

Figure S5. The TH5487 inhibitor interactions with OGG1 enzyme.

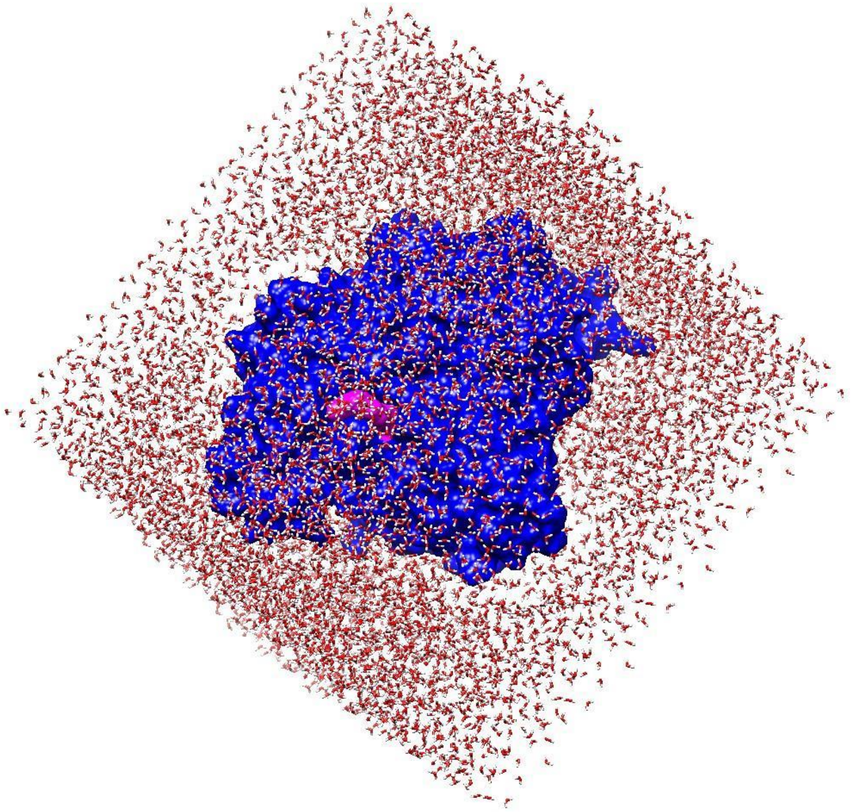

Figure S6. Submerged control- OGG1 complex (surface blue magenta) in TIP3P water box.
